# On Macroscopic Quantum Phenomena in Biomolecules and Cells: From Levinthal to Hopfield

**DOI:** 10.1155/2014/580491

**Published:** 2014-06-16

**Authors:** Dejan Raković, Miroljub Dugić, Jasmina Jeknić-Dugić, Milenko Plavšić, Stevo Jaćimovski, Jovan Šetrajčić

**Affiliations:** ^1^Faculty of Electrical Engineering, University of Belgrade, 11000 Belgrade, Serbia; ^2^Department of Physics, Faculty of Science, University of Kragujevac, 34000 Kragujevac, Serbia; ^3^Department of Physics, Faculty of Science, University of Niš, 18000 Niš, Serbia; ^4^Faculty of Technology and Metallurgy, University of Belgrade, 11000 Belgrade, Serbia; ^5^Academy of Criminalistic and Police Studies, 11000 Belgrade, Serbia; ^6^Department of Physics, Faculty of Sciences, University of Novi Sad, 21000 Novi Sad, Vojvodina, Serbia; ^7^Academy of Sciences and Arts of the Republic of Srpska, 78000 Banja Luka, Republic of Srpska, Bosnia and Herzegovina

## Abstract

In the context of the macroscopic quantum phenomena of the second kind, we hereby seek for a solution-in-principle of the long standing problem of the polymer folding, which was considered by Levinthal as (semi)classically intractable. To illuminate it, we applied quantum-chemical and quantum decoherence approaches to conformational transitions. Our analyses imply the existence of novel macroscopic quantum biomolecular phenomena, with biomolecular chain folding in an open environment considered as a subtle interplay between energy and conformation eigenstates of this biomolecule, governed by quantum-chemical and quantum decoherence laws. On the other hand, within an open biological cell, a system of all identical (noninteracting and dynamically noncoupled) biomolecular proteins might be considered as corresponding spatial quantum ensemble of these identical biomolecular processors, providing spatially distributed quantum solution to a single corresponding biomolecular chain folding, whose density of conformational states might be represented as Hopfield-like quantum-holographic associative neural network too (providing an equivalent global quantum-informational alternative to standard molecular-biology local biochemical approach in biomolecules and cells and higher hierarchical levels of organism, as well).

## 1. Introduction

*On Macroscopic Quantum Phenomena*. Initially,* quantum mechanics* appeared as a theory of* microscopic physical systems* (elementary particles, atoms, and molecules) and phenomena at small space-time scales; typically, quantum phenomena are manifested at dimensions smaller than 1 nm and time intervals shorter than 1 *μ*s. However, from the very beginning of the quantum mechanical founding the question of its* universality* was raised, that is, the question of general validity of the quantum-physical laws for* macroscopic phenomena*, usually treated by the methods of classical physics. In the history of quantum physics, and especially quantum mechanics, this question has been temporarily put aside for very different reasons, being considered as a difficult scientific problem. The situation is additionally complicated by the existence of different schools of quantum mechanics, arguing about physical-epistemological status of the so-called* collapse (reduction) of the wave function*. In this respect the situation is not much better today, and it can be said freely that the problem of universal validity of quantum mechanics is still open [1–15]. To this end, Primas [16] emphasizes the following.

“If we consider quantum mechanics as* universally valid* in the atomic, molecular, mesoscopic and engineering domain, then we have to require that a proper mathematical codification of this theory must be capable to describe all phenomena of molecular and engineering science. Already rather small molecules can have classical properties, so that a classical behavior is* not* a characteristic property of large systems. The existence of molecular superselection rules and of molecular classical observables is an empirically well-known fact in chemistry and molecular biology. The chirality of some molecules, the knot type of circular DNA-molecules, and the temperature of chemical substances are three rather different examples of molecular classical observables. Such empirical facts can be described in an ad hoc phenomenological manner, but it is not so easy to explain these phenomena from the first principles of quantum mechanics. A universally valid theory of matter has not only to describe but also to* explain* why the chirality of biomolecules (like the L-amino acids, the D-sugars, lipids or steroids) is a* classical* observable. The reality of this breakdown of the superposition principle of traditional quantum mechanics on the molecular level is dramatically demonstrated by the terrible Vontergan tragedy which caused many severe birth defects.”

Starting from the 1980s, mainly in the papers of Leggett [1, 2], a new period of investigation of quantum mechanical phenomena on the macroscopic level began. Namely, a clarification of the notions and planning of experimental situations for observing some physical effects started. The central problem in this respect is a notion of macroscopic differentiation of the states of quantum system whose quantum mechanical behavior is explored. More precisely, Leggett argues that the term* macroscopic quantum mechanical effect* must be related to macroscopically different states, that is, the system states (and observables) that carry macroscopic properties (and behaviors) of the system as a whole. These states (i.e., observables) must carry classical-physical behavior of the system as well; this poses a task for choosing physical conditions giving rise to observation of typical quantum effects related to these states. ^1^

Hence different kinds of macroscopic quantum phenomena: (i) the ones usually explored by the methods of (quantum) statistical physics and not related to macroscopically differentiated states (being classified as macroscopic quantum phenomena of the first kind, like solid state phenomena), and (ii) those ones regarding macroscopically different (differentiated) states (being classified as macroscopic quantum phenomena of the second kind and being interesting to us). Numerous different macroscopic quantum phenomena of the second kind, some of them belonging to the fast developing field of the quantum computing and information, unequivocally sharpen the overall problem of universal validity of quantum mechanics.

In the context of the macroscopic quantum phenomena of the second kind, we shall present a solution-in-principle of the long standing problem of the polymer folding (which was considered by Levinthal as (semi)classically intractable [17], as shortly reviewed below)—implying the existence of novel* macroscopic quantum biomolecular phenomena*, with far reaching implications.

*Levinthal Paradox Revisited*. Contemporary methods for calculation of conformational dependent chain properties are based on* thermodynamic* aspect of the problem, which explores* (semi)classically* the folding free energy landscape for protein with several successful attempts to model these processes* in silico* using molecular dynamics simulations with full atomic representation of both protein and solvent [18–22], producing continuous (semi)classical trajectories with the potential to connect* static structural snapshots* generated from experimental data. This is incorporated into the (semi)classical viewpoint that conformational changes of proteins, due to solvent, thermal, optical, and other influences of the environment, do not occur in a random way (e.g., movements of gas particles) but fold to their native conformation of deep global minimum in some (semi)classical funnel of low-energy conformations leading toward it [23]. Even in recently reported implementation of quantum annealing (on the programmable superconducting quantum device) for lattice protein folding problems, nothing quantum mechanical is implied about principles that govern the folding of protein chains [24] (rather quantum fluctuations are a tool used for solving the optimization problem of protein folding, considered classically intractable [25–27]).

Hence, these (semi)classical calculational methods do not describe properly* transitions* from one conformation to another, which is the* kinetic* aspect of the problem, exploring the conformation change of long flexible chain. This has been illustrated by Levinthal, who considered the probability of folding a protein molecule from coiled to native conformation [17]. Assume 2*n* torsional angles of an *n-*residue protein, each having three stabile rotational states; this yields 3^2*n*^ ≈ 10^*n*^ possible conformations for the chain; if a protein can explore new conformations in a random way at the rate that single bond can rotate, it can find approximately 10^13^ conformations per seconds; then the time *t* (s) required for a protein to explore all the conformations available to it is *t* = 10^*n*^/10^13^; for a rather small protein of *n* = 100 residues, one obtains *t* = 10^87^ s, which is immensely more than the apparent age of the universe (“Levinthal paradox”). Yet, according to experiments, proteins can fold to their native conformation in less than a few seconds [28].

It should be added that* (semi)classical* kinetic (nonstationary) predictions imply the continuous map/conformation change *k*_*i*_ → *k*_*f*_ which* requires* a sequence of *n* local* noncommuting* successive elementary transformations (local rotations of characteristic time *τ*_*o*_), with the time necessary for the net transformation much longer than characteristic time necessary for a local rotation (*τ*_*n*_ ~ *nτ*_*o*_ ≫ *τ*_*o*_) and the frequency of corresponding global transition much lower than the frequency of a local rotation (*f*_*n*_ ~ 1/*nτ*_*o*_ ~ *f*_*o*_/*n* ≪ *f*_*o*_)—strongly dependent on a degree of polymerization *n* (in clear* contradistinction* with the experimentally* observed* poorly dimensionally sensitive dispersion laws of the internal more or less delocalized quasiparticle excitations in any condensed state quantum system: electrons, phonons, and etc. [29]). Thus,* chain folding *based on* (semi)classical* (nonstationary) predictions cannot be considered kinetically understood; the same applies to* biomolecular recognition processes* based on* (semi)classical* selective ligand-proteins/target-receptors key/lock interactions.

## 2. Conformational Transitions in Biomolecules and Cells as Macroscopic Quantum Effects 

### 2.1. Quantum-Chemical Approach to Conformational Transitions in Biomolecules

**Figure 1:**
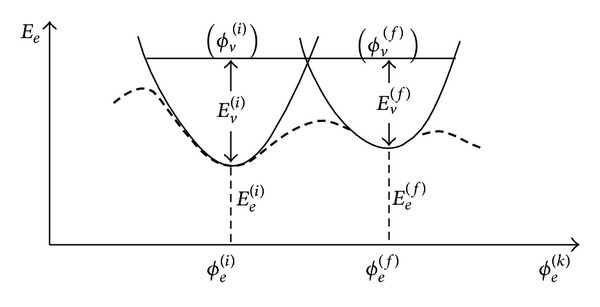
The (semi)classical problem of many-electron hypersurface *E*_*e*_(*ϕ*_*e*_^(*k*)^) as a potential energy for adiabatically decoupled Q1D vibrational and conformational system (with local minima as (semi)classical “positions”, i.e., many-atomic isomer configurations on many-electronic hypersurface (broken line in the figure))—not adiabatically well-defined when traversing between two adjacent local minima—is replaced in the framework of theory of nonradiative resonant transitions [30, 31] by better defined problem of two (virtually intersecting) isomeric many-electronic hypersurfaces (hyperparaboloids) serving as potential hypersurfaces for two vibrational (isomeric) problems (full line in the figure). In this approach, by time-dependent external perturbation of the isomer, at this very intersection, the conditions for electronic-vibrational nonradiative resonant transitions between the two isomers (*i*, *f*) are achieved: in the first approximation, the matrix element of dipole transition from *i*th to *f*th isomer is given by ***μ*** ^(*i*,*f*)^ ≈ ***μ***_*e*_^(*i*,*f*)^ *S*_*v*_^(*i*,*f*)^ + ***μ***_*v*_^(*i*,*f*)^ *S*_*e*_^(*i*,*f*)^. It is obvious that allowed transitions between isomeric states (*i*, *f*) are possible only for close states with nonvanishing electronic and vibrational dipole moments, ***μ***_*e*_^(*i*,*f*)^ and ***μ***_*v*_^(*i*,*f*)^ and nonvanishing electronic and vibrational overlap integrals *S*_*v*_^(*i*,*f*)^ and *S*_*e*_^(*i*,*f*)^ or in cascade resonant transitions between close intermediate participating isomeric states, which might be related to nondissipative polaron/soliton-like transport [32, 33]. Also, during these resonant transitions the perturbed biomolecular system is shortly described by quantum-coherent superposition $\left(\phi_{e}^{\left(i\right)}\phi_{v}^{\left(i\right)}\pm \phi_{e}^{\left(f\right)}\phi_{v}^{\left(f\right)}\right)/\sqrt{2}$, before its quantum decoherence into final electronic state *ϕ*_*e*_^(*f*)^ or into initial electronic state *ϕ*_*e*_^(*i*)^ (with subsequent deexcitations into lower vibrational states).

Within the framework of standard* quantum-chemical* Hamiltonian (including kinetic energies and Coulomb interactions of all biomolecular electrons and nuclei) and Born-Oppenheimer* adiabatic approximation* (of separated biomolecular electronic and vibrational degrees of freedom), the (semi)classical problem of many-electron hypersurface *E*_*e*_(*ϕ*_*e*_^(*k*)^) is replaced by better-defined problem of two (virtually intersecting) isomeric many-electron hypersurfaces (hyper-paraboloids) serving as potential hypersurfaces for two vibrational (isomeric) problems—within the theory of nonradiative resonant structural transitions [30]. In this approach, the conditions for electronic-vibrational nonradiative resonant transitions between the *i*th and*f*th isomeric states are possible only for close states with nonvanishing electronic and vibrational dipole moments and nonvanishing electronic and vibrational overlap integrals (cf.  Figure 1 and its caption for further explanation).

### 2.2. Quantum Decoherence Approach to Conformational Transitions in Biomolecules and Cells: From Levinthal to Hopfield

*Quantum decoherence approach* to conformational transitions [34–42] (cf. the Appendix) generally allows reproduction of both* existence and stability* of the (stationary)* conformations* and the* short time scales *for the quantum mechanical processes resulting effectively in (nonstationary)* conformational transitions* under external influences on the complementary environmental solution. This approach might also be applied to (nonstationary) mismatching-to-matching quantum mechanical* conformational transitions* in selective ligand-proteins/target-receptors key/lock* biomolecular recognition processes* under external (e.g., compositional/chemical, thermal/optical...) influences on the cell's complementary cytoplasmatic environment [36, 37, 41, 42].

In the context of existence and changes of conformations of biomolecules, it should be particularly pointed out that biomolecular operators of* Hamiltonian* $\overset{⌢}{H}$ (electronic-vibrational Hamiltonian, which includes operators of kinetic energies and all Coulomb interactions between the biomolecule electrons and nuclei in the center-of-mass coordinate system)* and conformations* $\overset{⌢}{K}$ (so-called “reaction (conformational)” coordinates of the nuclei, defining the biomolecule conformations) do not commute, $\left[\overset{⌢}{H},\overset{⌢}{K}\right]\neq 0$! Hence,* quantum-chemical approach* described in the previous section (with* simultaneously defined* energies and conformations of biomolecules)* is essentially (semi)classical*, and* it is only quantum decoherence that enables *appearance of* biomolecular conformational eigenstates* (labeled by upper index *K* in (1)) from the* biomolecular energy eigenstate of the isolated biomolecule *(labeled by upper index *E* in (1)) via* nonpotential interaction* of the biomolecular quantum system (QS) with its quantum environment (QE), when one of the biomolecular conformational *K*_*k*_ eigenstates is stochastically selected via quantum decoherence (QD)^2^ from the biomolecular initial many-electronic energy *E*_*e*_^(*i*)^ eigenstate of the isolated biomolecule (as only* self-Hamiltonian of the biomolecule* was switched-on initially, like a proper approximation when interaction with quantum environment might be accounted for via* potential term* of the self-Hamiltonian)^3^. It should be noted that the most probable biomolecular conformational eigenstate is the one labeled by *K*_*i*_, corresponding to biomolecular initial many-electronic energy *E*_*e*_^(*i*)^ (with the same index* i*, especially if it corresponds to biomolecular many-electronic ground state, in accordance with the usually adopted quantum-chemical computations within the framework of adiabatic approximation).

Subsequently, one of the stochastically* QD-selected biomolecular conformational K*_*k*_ *eigenstates* (*K*_*i*_, in Figure 1) might be* excited by nonstationary external perturbations* (photons…) into* some resonant electronic-vibrational energy eigenstate* (*E*_*e*_^(*i*)^ + *E*_*v*_^(*i*)^ = *E*_*e*_^(*f*)^ + *E*_*v*_^(*f*)^, in Figure 1), when* self-Hamiltonian of the biomolecule* is again a proper approximation (and interaction with quantum environment might be again accounted for via* potential term* of the self-Hamiltonian). Then, in subsequent quantum deexcitation/decoherence^3^ there are finally at least two possible biomolecular conformational eigenstates (as depicted in Figure 1): *K*_*i*_ related to biomolecular deexcitation back into initial many-electronic state *i* or *K*_*f*_ related to biomolecular deexcitation into final many-electronic state *f*.

And such fluctuations between eigenstates of energy and conformation of biomolecules are repeating

(1) |Φi〉QSE|Ψi〉QEE =∑jcj|Φj〉QSK|Ψj〉QEK  → QD |Φk〉QSK|Ψk〉QEK[⟶ρΦΨK]→ +ΔEexc  =∑lcl′|Φl〉QSE|Ψl〉QEE  → −ΔEdeec/QD |Φf〉QSE|Ψf〉QEE[⟶ρΦΨE]=⋯

and might be observed by applying methods of experimental macromolecular biophysics [43]—thus becoming a paradigm of macroscopic quantum phenomena of the second kind.

So, biomolecular chain folding in an open environmental solution might be considered as a subtle interplay between energy and conformation eigenstates of a biomolecule, governed by* local* quantum-chemical and quantum decoherence laws, and in this scenario the* Levinthal's paradox disappears* (cf. the Appendix for some aspects of* quantum decoherence scenario* of conformational transitions; also cf. footnote 5 therein for revealing* (semi)classical meaning *of the harmonic-like vibrating macromolecule conformations in the vicinity of local minimums of many-electron hypersurface).

**Figure 2:**
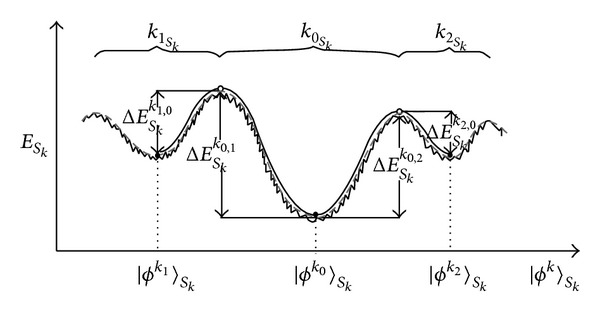
Schematic presentation of the memory attractors in the (many-electronic) energy-state (*E*_*S*_*k*__(*ϕ* ^*k*^)) hypersurface of the Hopfield-like quantum-holographic memory/propagator of the open macroscopic quantum (sub)system *S*_*k*_ of cell's particular spatial quantum ensemble of (noninteracting and dynamically noncoupled) *N*_*k*_ chemically identical proteins of *k*th type (and their corresponding biomolecular targets) [41, 42] in Feynman's representation [44]: *G*(*r*_2_, *t*_2_; *r*_1_, *t*_1_) = ∑_*i*_ *ϕ* ^*k*_*i*_^(*r*_2_, *t*_2_)*ϕ* ^*k*_i_*^(*r*_1_, *t*_1_) = ∑_*i*_ *A*_*k*_*i*__(*r*_2_, *t*_2_)*A*_*k*_i__*(*r*_1_, *t*_1_)*e* ^(*i*/*ℏ*)(*α*_*k*_*i*__(*r*_2_,*t*_2_)−*α*_*k*_*i*__(*r*_1_,*t*_1_))^. It should be pointed out that quantum decoherence presumably plays a fundamental role in biological quantum-holographic neural networks via energy-state hypersurface shape adaptation (in contrast to low-temperature artificial qubit quantum processors where it must be avoided until the very read-out act of quantum computation)—which implies that nature presumably has chosen an elegantroom-temperature solution for biological quantum-holographic information processing, permanently fluctuating between eigenstates of energy and conformation of the proteins of *k*th type (identical in their primary structure of the amino acids sequence) due to nonstationary environmental perturbations and subsequent decoherence by the environment, described by time-adapting density of conformational states (represented by corresponding depths of the minima in the figure above): ${\overset{⌢}{\rho }}_{S_{k}}^{k}\left(t\right)=\sum_{i}w_{k_{i}}\left(t\right)\left|{\phi^{k_{i}}\rangle }_{S_{k}}\langle \phi^{k_{i}}\right|,\sum_{i}w_{k_{i}}(t)=1$, of cell's biomolecular open macroscopic quantum (sub)system *S*_*k*_.

On the other hand, within an open biological cell, a system of (noninteracting and dynamically noncoupled) *N*_*k*_ proteins identical in their primary chemical structure (and their biomolecular targets) might be considered as corresponding* global* spatial quantum ensemble of *N*_*k*_ identical biomolecular processors, providing a spatially distributed quantum solution to corresponding single* local* biomolecular chain folding (and key-lock recognition process)—whose* time-adapting *density of conformational states ${\overset{⌢}{\rho }}_{S_{k}}^{k}(t)$ might be represented as* global* cell's* Hopfield-like quantum-holographic associative neural network* too [41, 42] (cf.  Figure 2 and its figure caption for further explanation). We hereby silently assumed* ergodic hypothesis*, that is,* near thermodynamic equilibrium* of the *N*_*k*_ proteins in their decoherence-selected (stationary)* conformations,* which is* not fulfilled in (nonstationary) conformational transitions* induced by* strong environmental interactions* (cf. the Appendix for more details on our* decoherence scenario*) which might occur* far from thermodynamic equilibrium* (as is the case in metabolic processes in biological cells [43]).

Or to generalize, a series of all *k* intracellular and extracellular environmentally driven (compositionally/chemically or thermally/optically)* local biochemically coupled reactions* might be* equivalently* considered as a series of all *k* corresponding intracellular and extracellular* global Hopfield-like quantum holographically coupled associative neural network layers*—providing an equivalent global quantum-informational alternative to standard molecular-biology local biochemical approach in biomolecules and cells (and higher hierarchical levels of organism, as well). ^4^

## 3. Discussion and Conclusion

*Biomolecules* in a living biological cell are subjected to* nonequilibrium* processes of huge complexity. Elaborate quantum mechanical descriptions of such processes are only a matter of recent considerations [45–47]. In this regard, the physical methods are a matter of intense current research [48, 49]. A fully developed quantitatively elaborate quantum mechanical background for such biological processes is yet a remote goal.

In the context of the* macroscopic quantum phenomena of the second kind* we hereby proposed quantum-chemical and quantum decoherence approaches to* biomolecular conformational transitions*, which cannot be considered kinetically understood based on (semi)classical predictions. Our qualitative proposal has a solid quantum mechanical basis of wide applicability: there are not any particular assumptions on the chemical kind, structure, or the initial state of the molecule or any assumptions on the chemical kind or on the initial state of the molecule's environment.

It seems that our matter-of-principle solution to the long standing* Levinthal paradox* offers a natural physical picture of a number of the important processes with biomolecules, including* chain folding* and* biomolecular recognition*. This offers a basis for some (semi)classical descriptions, such as the recent (also qualitative) proposal of Dill and Chan [23]. Actually,* quantum decoherence*is assumed to provide* (quasi)classical behavior* of the biomolecules conformation degrees of freedom, which can be further (semi)classically described to provide more details of the* biomolecule's conformation dynamics* in molecular biology and biochemistry.

Our model, *(A.2)*, is stochastic, not deterministic, and is sensitive to all allowed final conformations. Depending on the details of the physical model (initial state of the molecule, the kind and strengths of interactions with the solvent molecules, and the form of the energy landscape etc.), there is more than one possible final conformation in the sum equation *(A.2)* that in principle includes the initial conformation. Regarding the funnel landscape of Dill and Chan [23], a few scenarios are possible. For example, if the particle is in a thin local minimum, quantum tunneling can cause the highly semiclassical dynamic that is essentially described by Leggett [1] and qualitatively agrees with Dill and Chan [23]: the particle is expected quickly to go down the slope. On the other hand, for sufficiently deep local minimum, the particle can be trapped (e.g., in a metastable state), in which case the related conformation appears in the sum in *(A.2)*. If such local minimum is in the vicinity of the absolute minimum and the related conformations are practically indistinguishable, then our model predicts redefinition of the very concept of “native state (conformation).” In this case, “native state” does not refer to a single but to a set of close conformations of the molecule—again in accordance with the qualitative considerations of Dill and Chan [23] (and the references therein).

As another virtue of our decoherence model, we emphasize existence of a few different mechanisms for the externally induced conformational transitions. Those mechanisms (“channels”) are defined by local influence on certain subsystem without yet influencing the other subsystems (degrees of freedom) of the molecule. The subsystems of interest are conformational system, vibrational system (vibrational modes), the electrons system, and the local rotational degrees of freedom of the molecule. Realistic transitions can be assumed to be combinations of those local “channels” for conformational transitions. In principle, high precision and control of the molecular degrees of freedom can experimentally partially distinguish between the different channels. For instance, illuminating the molecule by the microwaves of the characteristic frequency ~10^9^ Hz should influence the local rotations in a molecule (with nonnegligible quantum tunneling between the allowed structural rotamers) without affecting the other degrees of freedom, while the infrared light of the frequency ~10^13^ Hz should influence the vibrations in a molecule with possible nonradiative resonant structural isomeric transitions (like in Figure 1, for transitions within the electronic ground state hypersurface). To this end, some basic details on the conformation-transitions mechanisms can be found in [32, 33, 39], respectively, while research is still in progress. Direct influence on the conformation, which is typically considered in the statistical (e.g., thermal equilibrium) approach, is rather subtle and is often described as a net effect mainly originating from a change of physicochemical characteristics of the solution (in the manner described by Anfinsen [24]) without resorting to an elaborate model yet; see, for example, [14, equation (3.164)].

Regarding the quantum ensemble prediction of our decoherence model (DM), *(A.2)* and the resembling Hopfield-like quantum-holographic neural network (HQHNN) bioinformational framework of the environmentally driven biochemical reactions on the level of open biological cell (Figure 2), there are several notes that might be added in proof: (i) biochemical reactions involve enzymatic processes, and enzyme's function is the DM conformational-adaptive one (so fundamentally every single biochemical reaction has bioinformational structure of HQHNN within the occupational basis of enzyme's conformational states, as an indicator of DM enzyme-mediated biochemical reactions); (ii) regarding all biochemical reactions of the particular type in the cell, a higher percentage of the functionally appropriate enzymes take their native conformation (usually the lowest energy state…) influenced by the proximity of the corresponding biomolecular substrate (key-lock enzyme-to-substrate DM conformational adjustments), but not the remaining percentage of those enzymes that are not yet in close interaction with their biomolecular substrates (which then occupy the remaining possible conformations as well, according to DM); (iii) in accordance with the previous point, all biochemical reactions of the particular type in the cell have bioinformational structure of HQHNN within the occupational basis of the corresponding enzyme's conformational states; (iv) taking into account other successive intracellular and extracellular environmentally driven biochemical reactions that are functionally interconnected with preceding biochemical reactions in the cell, they can also be successively presented in the bioinformational framework of HQHNNs within the occupational bases of conformational states of the corresponding enzymes involved; (v) since all these successive biochemical reactions are functionally interconnected, so are the successive HQHNNs in bioinformational framework within the corresponding enzymes' occupational bases (which may be presented in the form of Haken's multilevel synergetic neural network, composed of layers of the successive HQHNNs); and (vi) in such bioinformational framework of Haken's multilevel synergetic neural network, each of the successive HQHNNs layers representing corresponding intracellular and extracellular biochemical reactions has a formal Hopfield-like mathematical structure in the form of (nonmorphological/abstract) “formal neurons” massively interconnected by “formal connections” while the layers of HQHNNs would be mutually quantum holographically coupled via their “memory attractors” (i.e., their quantum-holographic memory states, within the occupational bases of conformational states of the corresponding enzymes involved).

Such a generalized bioinformational framework of Haken's multilevel synergetic neural network representing corresponding intracellular and extracellular biochemical reactions is in line with trends of modeling hierarchical information processing in higher cognitive processes [44] and might also provide possible missing downward causation control mechanism of morphogenesis and psychosomatics [41, 42, 50–52]. However, it should be noted that conditions for the above resemblance between quantum ensemble prediction of our DM and HQHNN frameworks are fulfilled* near thermodynamic equilibrium *(while predicted very nonstationary* conformational transitions*, induced by* strong environmental interactions *within the decoherence model (cf. the Appendix), might occur* far from thermodynamic equilibrium*).

## Figures and Tables

**Figure 3:**
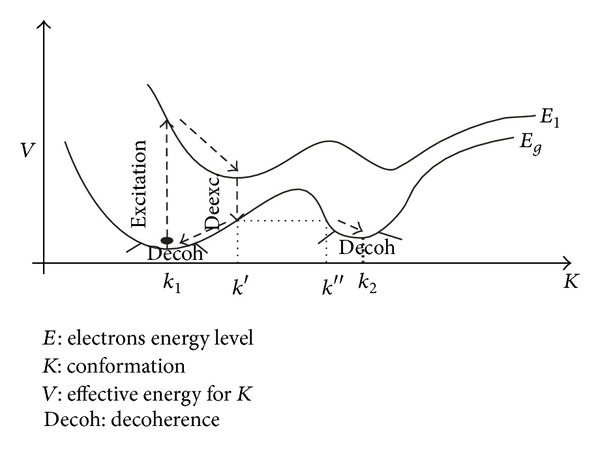
The black dot represents one-dimensional “particle” (macromolecular electronic-conformational-vibrational system) which is excited to the upper hypersurface (electronic excited state *E*_1_) and according to Ehrenfest theorem is descended down the slope towards the local excited state minimum, conformation *k*′. Then, the “particle” is deexcited to the lower hypersurface (electronic ground state *E*_*g*_) when according to Ehrenfest theorem a superposition of the two possible “particle” states depicted by *k*′ and *k*′′ is established, with subsequent coherent descent of the “particle” down both slopes of the barrier (which can be thought of as the interference of the two paths along the barrier walls) and final decoherence into local ground state minima *k*_1_ and *k*_2_ (with assumption that environmental influence dominates the dynamics in the vicinity of the local minima, corresponding to the acquired conformations *k*_1_ and *k*_2_—with their statistical weights being changed, as a net effect). For more details see [47].

## Endnotes

1. The Paradigm of the macroscopically differentiated states are the Gaussian states of the* center of mass* degreesof freedom of many-particle system. On the contrary, so-called* relative coordinates* (as observables) neither define macroscopically differentiated states nor carry classical behavior of the system, in any known physical theory or experimental situation.

2. In general, the stipulated* decoherence-preferred degrees of freedom* (biomolecular* conformations* in our case) are considered to be* accessible* (directly measurable) and therefore* objective for an environmental observer* (which is thus a part of the structure he observes). Are there some general rules and/or limitations for* all possible bi-partitions *QS_*k*_ + QE_*k*_ in the universe? It is not generally answered in QD theory and still needs* additional fenomenological assumptions* [13]. So, in the manner of* quantum chemistry* [29, 30] it might be plausibly proposed that many-atomic quantum systems QS_*k*_ are fenomenologically limited to the* structures with dynamically coupled electrons* considered as identical fermions, described by permutationally antisymmetric many-electronic eigenstates, which encompasses all existing molecules and electronic condensed state objects described by general quantum-chemical electronic self-Hamiltonian; in the case of the* structures with dynamically coupled identical bosons*, they are described by permutationally symmetric many-bosonic eigenstates and corresponding self-Hamiltonian.

3. In general, only* closed *composite system QS + QE is subject to the Schrödinger law (although this does not hold true separately for neither QS nor QE, as* open* quantum systems), with Hamiltonian $\overset{⌢}{H}={\overset{⌢}{H}}_{\text{QS}}+{\overset{⌢}{H}}_{\text{QE}}+{\overset{⌢}{H}}_{\mathrm{int}}$, where interaction Hamiltonian ${\overset{⌢}{H}}_{\mathrm{int}}$ depends on observables of both QS and QE. However, when ${\overset{⌢}{H}}_{\mathrm{int}}$ can be reduced to the “external field” its* potential term* $\overset{⌢}{V}$ can be added to ${\overset{⌢}{H}}_{\text{QS}}$, providing new self-Hamiltonian of the QS, dynamically decoupled from the observables of the QE, and then QS can be treated as the* closed* quantum system. This is the case in most situations in* quantum chemistry*, with Schrödinger's equation applied to the explored* closed* many-atomic quantum system with appropriate boundary conditions and adopted computational approximations (giving rise to* stationary* ground and excited electronic-vibrational energy* eigenstates* of all possible many-atomic* isomers*, corresponding to the minimum of the electronic potential hypersurface, depicted in Figure 1, for ground electronic and corresponding excited vibrational energy eigenstates) [29, 30]. It should be noted that Schrödinger's equation* cannot apply to nonstationary* excitations and relaxations of the many-atomic quantum system, not only in between different isomers but also within the same isomer—when quantum deexcitation/decoherence must apply to* nonpotential interaction* of the* open* many-atomic quantum system (*nondescribable* fully by its self-Hamiltonian) with its quantum environment (generally* field-related*, including vacuum) [12, 13].

4. The similar Hopfield-like quantum-holographic picture might also be applied to individual* acupuncture system* [41, 42] (with quantum-like macroscopic resonances, fenomenologically observed in microwave resonance therapy [50, 51], which implies corresponding bipartition QS_acu_ + QE_acu_, i.e., that acupuncture system has* macroscopic open quantum structure* with dynamically coupled electrons all along macroscopic network of acupuncture channels [41, 42, 50]). This then provides natural* quantum-informational framework* for psychosomatic medicine, that is,* quantum-holographic downward coupling* of the higher macroscopic quantum levels of* acupuncture system and its projection zones* (and presumably closely related* consciousness* [10, 41, 42]) with lower macroscopic quantum* cell's biomolecular level* changing the* expression of genes* (starting from the first fertilized cell division which initializes differentiation of the acupuncture system of nonthreshold electrical GJ-synapses (“gap-junctions”)) [41, 42, 52].

## Appendix

### On General Quantum Decoherence Framework for Macromolecular Conformations and Transitions

We assume that the macromolecule's environment selects the molecule conformation as the “pointer observable.” Following the general phenomenological results and understanding, we assume that decoherence takes place for virtually all kinds of macromolecules and the solvent environment* while* the composite system “macromolecule + environment” is not externally disturbed. This we call “*stationary state*,” for which we stipulate occurrence of decoherence, that is, the environment-induced classicality of the molecule conformation. Typically, the conformation transitions occur due to a severe external influence exerted on the macromolecular degrees of freedom and/or to the molecule environment. In effect, this external influence redefines the macromolecule environment and thereby also the influence exerted by the new environment to the macromolecule degrees of freedom. Such physical situations, which may take some time, we describe as “*nonstationary state.*” For the nonstationary state, no particular assumption is made. Rather, one may expect that the change in physical characteristics and state of the environment would typically violate the conditions assumed for the occurrence of decoherence.

In formal terms, the stationary state is defined by the conformation system “mixed” state,

(A.1) $$\begin{array}{c} \rho_{K}=\sum_{i}w_{i}{|k_{i}\rangle }_{K}\langle k_{i}|;\;\sum_{i}w_{i}=1, \end{array}$$

where states |*k*_*i*_〉_*K*_ represent the different conformational states. These (approximately orthogonal) states, |*k*_*i*_〉_*K*_, represent the preferred (semi)classical “pointer basis” states for the macromolecule conformation system. ^5^ Therefore, we stipulate the occurrence of decoherence as the fundamental quantum mechanical basis for the phenomenologically observed (semi)classical behavior of the macromolecules conformation stability.

On the other hand, as emphasized above, the conformational transitions occur due to a severe external influence. The related nonstationary state is defined by nonvalidity of (A.1) for the duration of the external influence. Intuitively, one may say that the external influence redefines the physical situation, the macromolecule is subjected to. In effect, the stationary state is disturbed, and there is not any semiclassical conformation state for the macromolecule. Formally, the conformation system is in state *ρ*_*K*_′, which cannot be presented by (A.1).

Of course, every external influence terminates and leaves the (redefined) system “molecule + environment” to relax, that is, to reach another stationary state with the final conformation state *ρ*_*K*_′′, which is representable in the form of (A.2). The point strongly to be emphasized is that it is highly unexpected that *ρ*_*K*_ = *ρ*_*K*_′′. That is, the final set of conformations need not be the same as the initial one, while for the conformations (i.e., states |*k*_*i*_〉_*K*_) common for *ρ*_*K*_ and *ρ*_*K*_′′, their statistical weights need not equal each other; *w*_*i*_ ≠ *w*_*m*_′′. In effect, the following transition of the conformation state occurred:

(A.2) ρK=∑iwi|ki〉K〈ki|→ nonstationary ρK′→ stationary ρK′′=∑mwm′′|km〉K〈km|; ∑iwi=1=∑mwm′′.

Duration of the whole dynamics presented by (A.2) is of the order of the time needed for the nonstationary state to terminate (note that decoherence, present for both stationary states, the most left hand and the most right hand sides of (A.2), is among the fastest physical processes known to date). So, in this scenario, the Levinthal's paradox disappears. Furthermore, as the concept of “trajectory” (in configuration space) is not well-defined quantum mechanically, the very basis of the Levinthal's paradox (i.e., sampling of trajectories in the configuration space) is absent in this quantum mechanical picture.

This general scenario has been analysed [46, 47] and a few possible scenarios of the external influence (i.e., of the nonstationary state) have been distinguished (see Figure 3 for a possible one).
